# Running a safe eye service for patients and personnel

**Published:** 2021-07-20

**Authors:** Astrid Leck

**Affiliations:** 1 Assistant Professor and Microbiologist: London School of Hygiene & Tropical Medicine, London, UK.

**The safety of staff members and the patients they care for is fundamental to high quality eye care delivery.**

**Figure:**
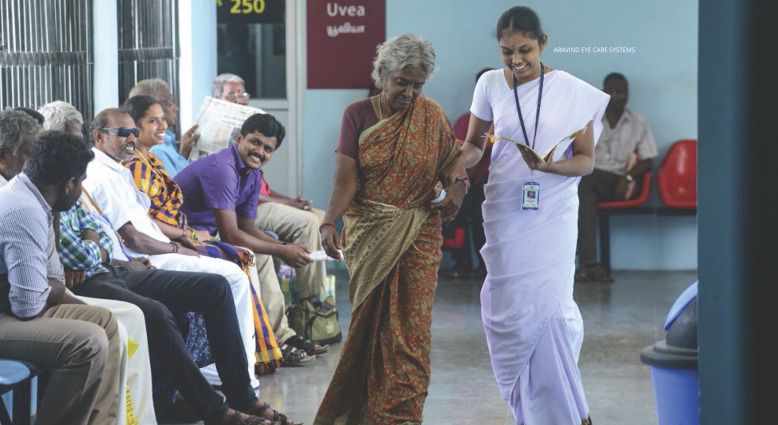
Safe eye care puts the wellbeing of staff members and patients first. **INDIA**

Safety is of paramount importance when delivering health care. Not only is it vital to keep patients safe during their journey to seek and receive medical care, it is equally important to protect health care’s most valuable resource, its personnel, or staff members.

Creating and maintaining a culture of safety within clinical settings should be a priority for health care leaders and managers; however, it is the responsibility of each and every member of a hospital or clinic’s workforce to adopt and champion safe practices.

In this issue, we explore what safe practice means specifically for eye care service providers by applying the fundamentals of safe practice both inside our institutions (by improving risk management, infection control, waste management, and safe prescribing of medication) and outside them – by keeping staff and patients safe during service delivery to local communities.

Creating a sustained safety programme can be challenging. Our authors offer practical tips and advice towards achieving this goal, mindful of the resource limitations many of us face.

Safety is intrinsically linked with the provision of high quality eye care; it should be an integral part of everything we do.

